# Determinants of unjustified cesarean section in two hospitals southwestern Ethiopia: retrospective record review

**DOI:** 10.1186/s13104-018-3336-3

**Published:** 2018-04-03

**Authors:** Tensay Kahsay W/gebriel, Tegene Legesse Dadi, Kebadnew Mulatu Mihrete

**Affiliations:** 1 School of Nursing, College of Health Sciences, Mekelle University, Mekelle, Ethiopia; 2 Department of Public Health, College of Health Sciences, Mizan-Tepi University, Mizan, Ethiopia

**Keywords:** Cesarean section, Determinants, Ethiopia

## Abstract

**Objective:**

The study aimed to identify determinants of unjustified cesarean section in two hospitals southwestern Ethiopia using retrospective record review from January 2015 to January 2016.

**Result:**

A total of 727 charts were included in the analysis. About 25% of the study participants had delivered by cesarean section in 1 year. Antenatal care visit (AOR = 0.003, 95% CI 0.00–0.07), labor abnormality (AOR = 10.1, 95% CI 4.61–22.1), and post term pregnancy (AOR = 10.6, 95% CI 4.85–23.1) were significantly associated with cesarean section when compared to their respective counterparts.

## Introduction

Giving birth in its natural process is likely to introduced risks for the mother and/or her fetus, irrespective of the route of delivery (cesarean or vaginal delivery) [1]. This declares that the unreserved efforts of health professionals to minimize pregnancy and delivery outcomes with less likely complications in the mother and fetus. To make this cesarean section delivery is one of the obstetric managements introduced [2]. Cesarean section (C/S) is a term commonly used in obstetrics to describe the delivery of a viable fetus through an incision in the abdominal wall (laparotomy) and the uterus (hysterotomy) [3]. Even if C/S delivery can be life-saving for the mother and/or fetus, the quick rise in the rate of C/S delivery without indication to decrease in maternal and neonatal morbidity is overused [4].

Currently, World Health Organization (WHO) stated that there is no additional health benefits associated with a caesarean section rate above 15% [5]. Although WHO estimates the levels of C/S between 10 and 15% were stated as high but conventional at the time, average C/S rates in most developed regions were now exceeded 20% [6]. Increasing caesarean section rate is an issue of public health concern globally for last 30 years; its use has increased since 1970 to a level that is medically unjustified. Thus bringing negative economic and health related repercussions [7]. The study aimed at to identify determinants of unjustified cesarean section in two hospitals southwestern Ethiopia using retrospective record review from January 2015 to January 2016.

## Main text

### Methods

Institutional based retrospective chart reviews was done to analyze the data from maternal charts registered in the logbook of obstetrics and gynecology units for different health services in Mizan-Tepi university Teaching and Bonga general hospital from January 2015 to January 2016, southwest Ethiopia. The hospitals provide different health care services for about 5 million populations.

Data were collected using questionnaire which was adapted from Ethiopia maternal death surveillance guideline [8]. The tool contains of socio-demographic data, obstetric and delivery history, presence of co-morbidities, and birth outcome. Three midwives and nurses were recruited as data collectors and trained for 2 days prior the data collection time about the objective of the study, ethical considerations and how to collect necessary data from the maternal chart. Overall the data collection process was supervised by two midwives and one registered nurse. In addition, the principal investigators have closely monitored and followed the data collection process.

A total of 763 maternal charts which were registered in obstetrics and gynecology logbook for different maternal care service in 1 year in the two hospitals were reviewed. Of which, 36 charts were incomplete, so discarded from the analysis. Finally 727 maternal charts were included in the analysis.

After data collection, questionnaires were coded by the principal investigators. Data were entered to Epi Data version 3.1 and then exported to STATA version 13.1 for analysis. Descriptive frequency was carried out to check for any entry errors and corrections was made for identified errors.

First bivariate analysis was carried out, and then multivariate logistic regression analysis was used to identify the association between dependent and independent variable and the confounder. Confidence interval of 95% was used to see the precision of the study and the level of significance was taken at p ≤ 0.05. Results were presented in text, tables and figures.

Ethical approval was waived from the college of health sciences Mizan-Tepi university ethical committees according the university ethical clearance rule and regulation. Then, approbation letter was also obtained from the hospital administers and Bench-Maji and Kefa Zonal Health Offices. The maternal information collected from the charts was kept confidential throughout the study.

### Result

**Fig. 1:**
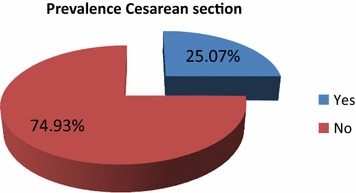
Overall cesarean section performed among women attending health service utilization in Mizan-Tepi university teaching and Bonga general hospitals, 2016 (n = 727)

Out of 763 maternal charts reviewed, 727 were included in the analysis. Majority 567 (77.99%). of the participants were aged at 20–34 years. about 507 (69.7%) study subjects had ANC visit, of which, 252 (50%) had 4 and above ANC visits. From all, 99 (14%) mothers had greater than 24 h length of labor, while 137 (19%) had obstetric complication (PROM, malpresentation, oligo and poly hydamnos). Women who were referred from other facilities accounts 247 (34%). In 8 (1%), 11 (1.5%) and 29 (4%) of the participants had previous C/S delivery, preeclampsia, and vaginal bleeding (Table 1). The overall caesarean section use in the hospitals in this study was 182 (25.07%) (Fig. 1).

**Table 1:** Obstetrics findings of women attending health service utilization in Mizan-Tepi university teaching and Bonga general hospitals, 2016

Variables	Frequency	Percent
Age in years		
< 20	109	15
20–34	567	78
> 34	51	7
No. of gravidity		
≤ 2	484	66.57
3–4	154	21.18
≥ 5	89	12.24
No. of parity		
≤ 2	590	81.16
3–4	97	13.34
≥ 5	40	5.5
ANC attendance		
No	220	30.26
Yes	507	69.74
No of ANC visits		
1	135	26.63
2–3	120	23.67
≥4	252	49.7
Referral to the hospitals		
No	480	66.02
Yes	247	33.98
Length of labour (h)		
< 24	627	86.36
≥ 24	99	13.64
Labour abnormality		
No	552	76.03
Yes	174	23.97
Birth attendant		
Doctors	125	17.22
Midwives	496	68.32
Master in emergency obstetrics	105	14.46
Weight of new born (kg)		
< 2.5	29	3.99
≥ 2.5	698	96.01

*ANC* antenatal care, *APH* antepartum hemorrhage, *C/S* cesarean section

#### Determinants of unjustified cesarean section

The result of bivariate logistic regression analysis showed that, age, ANC visit, gravidity, referral history, obstetric complication, post term pregnancy, labour abnormality, and the weight of fetus were associated with cesarean section (Table 2). However, on multivariable variable logistic regression analysis ANC visit, referral history, labour abnormality, obstetric complication, weight of fetus, and post term pregnancy were found to be significantly associated with cesarean section as clearly depicted on table below (Table 2).

**Table 2:** Determinants of cesarean section among women attending health service utilization in Mizan-Tepi university teaching and Bonga general hospitals, 2016 (n = 727)

Variables	Frequency (%)	Crude OR (95%)	Adjusted OR		95% CI	p-value
Age category						
< 20	109 (14.99)	Reference group				
20–34	567 (77.99)	0.370 (0.307, 0.445)		3.843	(1.246, 11.86)	0.019
> 34	51 (7.02)	0.700 (0.401, 1.223)		4.514	(0.807, 25.24)	0.086
No. of gravidity						
≤ 2	484 (66.57)	0.295 (0.238, 0.365)	0.153	2.042	(0.767, 5.441)	0.153
3–4	154 (21.18)	0.426 (0.302, 0.601)	0.950	1.034	(0.357, 2.995)	0.95
≥ 5	89 (12.24)	Reference group				
ANC attendance						
No	220 (30.26)	Reference group				
Yes	507 (69.74)	0.281 (0.228, 0.347)		0.003	(0.000, 0.07)*	< 0.001
Number of ANC visits						
1	135 (26.63)	0.288 (0.192, 0.433)		1.215	(0.592, 2.49)	0.596
2–3	120 (23.67)	0.237 (0.151, 0.37)		0.744	(0.365, 1.52)	0.417
≥ 4	252 (49.7)	Reference group				
Referred						
No	480 (66.02)	Reference group				
Yes	247 (33.98)	0.388 (0.294, 0.512)		0.41	(0.19, 0.86)*	0.019
Length of labour (h)						
< 24	627 (86.36)	Reference group				
≥ 24	99 (13.64)	0.800 (0.538, 1.189)		1.172	(0.512, 2.68)	0.706
Labour abnormality						
No	552 (76.03)	Reference group				
Yes	174 (23.97)	1.260 (0.934, 1.699)		10.089	(4.61, 22.1)*	< 0.001
Obstetric complications						
No	590 (81.16)	Reference group				
Yes	137 (18.84)	1.894 (1.330, 2.696)		10.579	(4.9, 23.06*)	< 0.001
Post term pregnancy						
No	704 (96.84)	Reference group				
Yes	23 (3.16)	22.00 (2.96,163.21)		10.000	(1.14, 87.9)*	0.038
Placenta previa						
No	716 (98.49)	Reference group				
Yes	11 (1.51)	1.200 (0.366, 3.932)		0.632	(0.04, 10.13)	0.0746
APH						
No	698 (96.01)	Reference group				
Yes	29 (3.99)	0.933 (0.451, 1.934)		1.382	(0.116, 16.4)	0.798
Multiple gestation						
No	697 (95.87)	Reference group				
Yes	30 (4.13)	0.071 (0.017, 0.300)		0.078	(0.008, 0.8)*	0.028
Weight of new born (kg)						
< 2.5	29 (3.99)	Reference group				
≥ 2.5	698 (96.01)	0.351 (0.296, 0.415)		5.598	(0.35, 90.1)*	0.024

*ANC* antenatal care, *APH* antepartum hemorrhage, *C/S* cesarean section

* Shows significantly association at 95% CI, p-value of < 0.05

There is strong association between ANC visit and cesarean section. Those who had ANC follow up were 99% less likely to have cesarean section than their counterparts (AOR = 0.003, 95% CI 0.00–0.07). And those who were referral to come to the hospitals were 3 times increased the odds of having cesarean section than their counterparts (AOR = 3, 95% CI 0.195–0.862) (Table 2).

Labour abnormality was also significantly associated with cesarean section. Women who had labour abnormality were 10 times at increased risk of cesarean section when compared to their counterparts (AOR = 10.1, 95% CI 4.61–22.1). Similarly those who had post term pregnancy were 10 times more likely to be at risk for cesarean section than their counterparts (AOR = 10.6, 95% CI 4.85–23.1) and Women who were multiple gestations where 92% less likely to have unjustified C/S compared to their counterparts (Table 2).

Infant weight greater than 2.5 kg were 5.6 times more at risk for cesarean section than those whose infant weight less than 2.5 kg (AOR = 5.6, 95% CI 0.35–90.11). Significant association was observed between obstetric complications and C/S. That is those who had obstetric complications 10 times odds of cesarean section than their counterparts (AOR = 10.5, 95% CI 4.85, 23.1) and women waving length of labour, placenta previa and APH were not significantly associated with unjustified C/S (Table 2).

### Discussion

In this study the proportion of women who underwent C/S delivery in the study hospitals was 25%. This result is inline with findings showed in other parts of Ethiopia [9–11]. On the other hand, it was less than the study that was conducted in Ethiopia [12] and outside of Ethiopia [13, 14]. However, it is unjustified than World Health Organization (WHO) recommendation which is 5–15%. That is the frequency of cesarean section depends on the socio-demographic pattern, hospital policies regarding management of cases of dystocia, mal presentation and previous cesarean section, and consideration of maternal choice and wishes. This magnitude C/S may be attributed to high number of referred cases and the catchment areas with the two hospitals.

The study also showed that ANC visit has significant association with cesarean section. That is those who had ANC visit were 99% less likely for cesarean section than their counterparts. This is similar with the finding of [9]. It also showed that, those who had obstetric complication, like APH, preeclampsia, and multiple gestations were 10 times increase the odds of having cesarean sections when compared with those who did not have obstetric complication. This finding is in line with the findings that was done in other parts of Ethiopia [9, 15] and outside of Ethiopia [16–18].

There is also a significant statistical association between labour abnormality and cesarean section. That is women who had labor abnormality such as mal presentation were 10 times increase the odds of having cesarean section than those did not have labour abnormality. This finding was in agreement with the study findings that were done inside country [9, 10] and outside of the country [18]. And those women who had baby weight greater than 2.5 were 5.6 times increase the likely hood of having cesarean section than their counter parts. This finding is in line with other study findings which were done in other parts of Ethiopia [10, 11] and outside [19]. This might be explained by when the weight of the fetus becomes large, it might lead to cephalo-pelvic disproportion which is the major indication for cesarean section.

Those women who come by referral from different health institutions were 59% less likely for C/S than those who did not haves. The current finding is different from findings that were done in Attat Hospital, Ethiopia, which implicates those referred women were most likely to C/S [9]. It might be explained by those who were referred might be self-referral for better investigation and might get early intervention other than C/S augmentation and instrumental delivers most helpful to minimize C/S.

### Conclusions and recommendations

The study showed that the overall cesarean section performed in 1 year was 25% in the study hospitals. It is above 15%, which is recommended by WHO for developing countries, so this indicates unjustified use of the service in the study areas. If it is not seen critically, the rate might reach intolerable levels. It is fact; Cesarean section that is performed for appropriate medical or obstetric indications is life saving for both the mother and the new born. But the unjustified use of C/S, does not mean that the perinatal outcome is improved rather it has risks for the mother and the neonate. The study also showed that post-term pregnancy, labour abnormality, baby weight, ANC visit, and referral history were determinants to unjustified cesarean section in the study area.

Health extension worker should work in strengthen manner in the community to increase the uptake of ANC utilization. In order to decrease high use of C/S, each woman should be carefully assessed and followed to identify the option for vaginal delivery.

## Limitation of study

- Since this is secondary data it has limitation related with missing of information or pertinent variables.

## Abbreviations

ANC: antenatal care
APH: antepartum hemorrhage
AOR: adjusted odds ratio
CI: confidence interval
C/S: cesarean section
CPD: cephalic pelvic disproportion
OR: odds ratio
PROM: premature rupture of membrane
SD: standard deviation
SNNPR: Southern Nations, Nationalities, and Peoples Region
WHO: World Health Organization
